# LONG-TERM OUTDOOR AIR POLLUTION CONCENTRATIONS AND COGNITION: FINDINGS FROM THE HCAP SUBSTUDY OF ELSA

**DOI:** 10.1093/geroni/igae098.1251

**Published:** 2024-12-31

**Authors:** Giorgio Di Gessa

**Affiliations:** University College London, London, England, United Kingdom

## Abstract

**Background:**

Although air pollution has been associated with cognitive impairment, several key details of these relationships remain unknown. This includes understanding how different pollutants and their emission sources impact the brain as well as the particular aspects of cognition that are susceptible to pollution. In this study, we consider the associations between 8 years of exposure to different air pollutants (including total PM2.5 and PM2.5 from different emission sources, and NO2) with different domains of cognitive performance among older people.

**Methods:**

We used data from the Harmonized Cognitive Assessment Protocol (HCAP) sub-study of the English Longitudinal Study of Ageing (2018, N~1,200). This study measured a range of key cognitive domains affected by cognitive ageing, including attention, memory, executive function, language, and visuospatial function. Outdoor concentrations of each pollutant for all HCAP respondents’ residences were assessed since 2010. Averages and group-based trajectory modelling were then used to summarise long-term air pollution exposures and their relationships with cognition After adjustment for key personal and neighbourhood confounders.

**Results:**

Preliminary results suggest that respondents with higher residential levels of air pollution reported poorer cognitive function, especially for verbal fluency and executive function. Particularly, we found sustained high levels of NO2 to be associated with poorer cognition, even when baseline levels of cognition were accounted for (B=-1.75, 95%CI=-3.43;-0.06). Regarding PM2.5, our results suggest that emissions from industry are particularly detrimental to cognition.

**Conclusions:**

Efforts to reduce levels of exposure to air pollution should continue as this is a risk factor for poorer cognitive performance.

